# Valorisation of Aggregate-Washing Sludges in Innovative Applications in Construction

**DOI:** 10.3390/ma17194892

**Published:** 2024-10-05

**Authors:** Yury Villagran-Zaccardi, Francisca Carreño, Line Granheim, Antonio Espín de Gea, Ulf Smith Minke, Stefania Butera, Elena López-Martínez, Arne Peys

**Affiliations:** 1 Materials and Chemistry Unit, Flemish Institute for Technological Research (VITO), 2400 Mol, Belgium; arne.peys@vito.be; 2 Centre for Technology for Marble, Stone, and Materials (CTM), 30430 Cehegín, Spain; paqui.cf@ctmarmol.es (F.C.); antonio.espin@ctmarmol.es (A.E.d.G.); elena.lopez@ctmarmol.es (E.L.-M.); 3 Building and Construction, Danish Technological Institute (DTI), 2630 Taastrup, Denmark; ling@teknologisk.dk (L.G.); usm@teknologisk.dk (U.S.M.); stbu@teknologisk.dk (S.B.)

**Keywords:** mining and quarrying waste, circularity, End-of-Waste, secondary filler, supplementary cementitious material, lightweight aggregates, 3D printing

## Abstract

The valorisation of sludges from aggregate production into construction materials is required for full circularity in mining waste management. This study explores valorisation pathways, relevant regulatory frameworks, and End-of-Waste (EoW) criteria for specific settings in Spain and Norway. The explored valorisation routes involved the production of filler, supplementary cementitious materials (SCMs), and lightweight aggregates (LWAs) for the production of cement-based products, and precursors for 3D printed construction material. The sludge from Norway revealed a non-polluted stream and a stream contaminated with organic phases and clays. Sludge-based filler proved suitable in concrete production with contents of up to 40% of total binder, providing adequate consistency and cohesion. However, clays in the sludge increased the demand for water and superplasticizer. Clay contents were still insufficient for the applications as SCMs, as the calcined sludge demonstrated limited reactivity. The application to produce LWAs was promising, but further microstructure optimization is still required. The clay content was also relevant for the sludge from the site in Spain, as it provided 3D printing mixes with good plasticity. The dosage optimization still required the addition of enzymes, limestone, and natural fibres to improve cohesion, workability, and resistance to the cracking of the 3D printing mix.

## 1. Introduction

The mining and aggregate industries generate significant amounts of waste, which are typically used in low-value backfilling or landfilled. Some of the waste fractions with the largest potential are washing-sludges. Such secondary resources have abundant availability (compatible with the size of demand in the construction industry) and a consistent mineral composition, and are cost-effective. Given the close relationship between the mining and construction industry, using these resources as secondary raw materials in construction makes a great deal of sense.

Considering the current and future demand for construction materials, four relevant technologies are identified for valorising mineral waste streams as precursors: (1) the production of inert filler for concrete, (2) the production of a supplementary cementitious material, (3) the production of lightweight aggregate, and (4) 3D printed mixes. Each of these strategies implies particular challenges and potential, for which some of the most important factors are the particle size distribution and content of clay in the material.

The application as a filler in the production of self-compacting mixes is a straightforward valorisation route that requires a substantial amount of fines. Using secondary fines reduces the demand for virgin resources such as limestone. Initially, the inclusion of filler might be assumed as a simple dilution effect on the performance of a concrete mix, but the impact on the concrete performance is tied to the specific properties of the filler. The dilution effect is not a critical issue for the particular case of self-compacting mixes as the high volume of fines is intended to provide the necessary fluidity and cohesion [1]. Therefore, the assessment is more complex than a simple examination of the dilution effect.

The presence of contaminants like clay can affect the mix properties. Adequate workability can be achieved by a rational design that subsequently optimises the water-to-powder ratio, filler-to-cement ratio, and superplasticiser content of the mixes [1]. In some cases, clay contents may reach moderate contents in the sludges (>15 wt%) with potential effects on water demand and cohesion [2,3,4,5,6]. The technical feasibility of such an approach may require reducing the impact of clay content for improved performance and cost reduction [1]. For the washing sludges, the content of clay may also contribute with increased viscosity in spite of the associated increase in superplasticiser demand [6]. Lei and Plank [7] found that the dispersing ability of most polycarboxylates is much impeded by montmorillonite impurities. Also, Xiong et al. [8] found that montmorillonite, illite, and kaolinite can double or triple the demand for polycarboxylate for maintaining the fluidity. An increase in viscosity is inevitable. A derived advantage of this increased viscosity is the potential capacity for increasing the maximum aggregate size (usually limited to 12–19 mm) in self-compacting concrete. If a stable mix with a larger maximum aggregate size can be produced, less paste will be needed, and more eco-efficient mixes will be obtained as a consequence. Despite the fact that much research has been conducted using pure clays, there is no clarity on the effects of sludges containing various types of clay as minor constituents.

The moderate content of clay opens the possibility of additional value for sludges using a thermal treatment. A calcination process at 600–900 °C increases the reactivity as supplementary cementitious material (SCM) by dehydroxylating and amorphising clay minerals or hydroxylated feldspathoids [9]. The calcination of sludges is therefore relevant when a sufficient amount of such phases is present. Most literature considers kaolinitic clays to produce SCMs, often in combination with limestone to produce LC^3^ cement [10]. Sludges containing kaolinite are therefore excellent candidates for beneficiation as SCM using the calcination process. A substantial amount of kaolinite has been reported in other mineral wastes such as coal tailings and bauxite tailings, and has therefore often delivered sufficient reactivity as SCM [11,12]. Although metakaolin is superior in reactivity, other clays can also provide sufficient reactivity. Montmorillonite and other smectites or illite have shown adequate reactivity after calcination [13,14,15] and even residues containing them—such as dredged sediments—can reach 250–270 J/g in an R^3^ test after flash calcination [16]. Similarly, hydroxylated feldspathoids (e.g., sodalite or cancrinite) have been shown to provide reactivity to bauxite residue [17].

An alternative thermal treatment is bloating, to produce lightweight aggregates similar to expanded clays. The chemical composition of the feed is crucial to control the melt formation as a function of temperature, melt viscosity, and bubble formation. Compositional ranges were established by Riley [18], which mainly indicates the importance of having sufficient SiO_2_ and some Al_2_O_3_ and flux elements (Fe, Na, K, Ca, and Mg). An alternative method to produce lightweight aggregates for materials without the suitable chemical composition is cold granulation using a cementitious binder. In this process, materials with good properties as filler are preferred. The process has therefore been used to produce aggregates from calcite-rich mine tailings [19], which was applied at pilot scale and implemented in concrete mixtures [20]. When using tailings with a minor reactivity as SCM, cold granulation using an alkaline activator has been tested as well [21,22].

Enriched clay may be used in mortars as a promising option for 3D printing due to clay’s renewability, abundance, affordability, and historical use in construction [23]. With a relatively low carbon footprint [24] and favourable thermal/acoustic properties [25], clay mortars offer sustainability benefits crucial for addressing global construction challenges. Washing sludges containing a high content of clays sourced from quarries dedicated to the extraction of aggregates can be used as byproducts in the production chain towards cement-free 3D printing applications. Aspects such as strength development and shrinkage are some of the main challenges that need to be addressed [24,25].

## 2. Regulatory Framework and Relevance of End-of-Waste (EoW) Criteria

### 2.1. Introduction to Mining Waste and Legislation for Waste Management

Waste from extractive industries refers to residual materials resulting from the prospecting, extraction, treatment, and storage of mineral resources and the working of quarries [26]. Mining waste thus covers a wide range of materials, from natural materials that have undergone minimal processing, to materials that have been more extensively processed during stages of mineral and metallurgical refinement. These materials may contain various inorganic and organic substances, reflecting the diverse physical and chemical characteristics of the materials involved. For the applications described in the previous section, the content of clays is demonstrated to be one of the main aspects to consider. Some particular cases may also require looking into heavy metal contamination levels, which imply a potential burden for applications in construction. These variations in mineral composition can lead to different potential environmental impacts, emphasising the importance of the proper handling and management of the waste streams generated.

The management of waste from mining and quarrying (M&Q) activities within the European Union is governed by Directive 2006/21/EC on the management of waste from extractive industries, also known as the Mining Waste Directive [26]. The directive provides specifications and requirements for the management of waste generated from mining activities, with the overall aim to minimise environmental impacts and enhance sustainable mining practices. The directive is adopted into national legislation by each Member State, which ensures that the principles and objectives of the directive are integrated into their legal systems, thereby facilitating the safe and environmentally sound management of mining waste within their jurisdictions.

In recent years, there has been a growing interest in the enhanced recovery and valorisation of waste, including waste from mining activities. By recovering valuable materials from waste, the demand for virgin resources can be reduced, thus minimising the environmental impacts associated with mining. Additionally, efficient waste recovery can help mitigate the potential adverse effects of waste disposal, such as soil contamination and water pollution. Economically, enhanced waste recovery can contribute to the circular economy by its potential to transform waste into valuable secondary raw materials. The valorisation path to produce SCM may even help reduce the consumption of carbon intensive clinker in the production of cement-based materials. This not only generates new revenue streams but also reduces the costs associated with waste management and disposal. Furthermore, advancements in technology have made it more viable to extract valuable metals and minerals from mining waste, thereby enhancing the profitability of recovery processes. The valorisation of mineral wastes aligns with the goals of the EU’s Green Deal and Circular Economy Action Plan by conserving natural resources, reducing environmental impacts, and promoting economic growth through sustainable practices [27,28]. Consequently, Member States can support the EU’s ambitious targets and play a central role in driving the green transition.

Waste resulting from mineral processing activities, such as washing sludges, can potentially pose challenges for waste management and later application as recycled materials due to their fine particle size and potential chemical contaminants. However, the potential for these materials to be recycled as secondary raw materials is gaining increased attention. This interest is driven by innovative projects like ROTATE [29], which aims to deliver circular solutions for waste valorisation by creating a symbiosis between the M&Q industry and the construction sector. Central to increasing material circulation is the incorporation of the End-of-Waste (EoW) concept, which provides a structured framework for determining when waste ceases to be waste and can be regarded as a secondary raw material or product to be freely traded on the open market [30]. By adhering to EoW criteria, one can ensure compliance with relevant legislation, meet specific product standards and align with market requirements, which is essential for gaining regulatory approval and public acceptance for the successful integration of recycled materials into construction applications.

### 2.2. Relevance of End-of-Waste (EoW) in Management of Mining Waste

The concept of End-of-Waste is introduced in Directive 2008/98/EC [30], commonly known as the Waste Framework Directive (WFD). The WFD sets forth specific conditions under which, at the EU level, waste can achieve End-of-Waste status. According to Article 6 of the WFD, waste may cease to be waste when it complies with specific criteria developed in accordance with the following conditions:

- The substance or object is commonly used for specific purposes.
- A market or demand exists for such a substance or object.
- The substance or object fulfils the technical requirements for the specific purposes and meets the existing legislation and standards applicable to products.
- The use of the substance or object will not lead to overall adverse environmental or human health impacts.

The rationale for establishing EoW criteria is to facilitate and promote recycling, while ensuring high environmental protection and economic feasibility. By clearly defining when a waste ceases to be waste and becomes a secondary product, recycling processes can be streamlined to comply with the defined requirements.

The lack of uniform regulations across EU Member States creates legal uncertainties in waste management, particularly affecting the recycling and reuse of secondary materials. This inconsistency complicates trade between Member States, as different countries may have incompatible regulatory frameworks. Consequently, producers and consumers often limit their activities to national markets to avoid legal and economic risks associated with the status of the material. Establishing clear, EU-wide criteria defining when waste ceases to be waste could thus facilitate the expansion of the market for recovered materials. A general methodology for the development of EoW criteria for specific waste streams was published by the Joint Research Centre (JRC) in 2009 [31]. The methodology provides a comprehensive framework that includes the identification and assessment of the material’s properties, market potential, relevant legislation, and stakeholder interests.

Given the diverse nature of waste materials, EoW criteria are tailored to individual waste streams and materials. This specificity is essential to address the different environmental concerns associated with each material and to ensure that the criteria are both relevant and effective. Currently, EoW criteria have only been developed at the European level for a limited number of material streams (e.g., glass cullet and certain types of scrap metal). This can be attributed to the inherent complexity and diversity of waste and materials. Furthermore, the establishment of EoW criteria involves extensive research, stakeholder engagement, and regulatory alignment between Member States, often demanding significant time and resources. However, the End-of-Waste concept can also be applied at the national level, allowing each Member State to develop criteria that address their specific waste management needs and regulatory contexts. This national-level application allows for adaptation to local conditions and enables a more targeted approach to waste valorisation.

### 2.3. Regulatory and Policy Challenges and Opportunities for Waste Valorisation

For the study sites in Norway (Velde), and Spain (Hormisoria), the washing residues are shown to have chemical and phase compositions related to the primary target materials being washed (aggregates and sand), albeit with a significantly smaller particle size. The fines tend to concentrate some phases from the original source material, especially those that are softer and more easily milled during the processing (e.g., clay material). Although the same phases are normally present in the coarse aggregates and sludges, the relative content of each of these varies quite significantly. Due to the fine particle size of the sludges (<63 μm), the non-polluted residues are typically used in low-value applications such as backfilling, primarily because higher-value applications are not technically or economically feasible at present. With the presence of contamination in the sludges, as is the case for the washing residue from the treatment of polluted excavated material in Norway, the landfilling of the material is more likely, depending on national legislation.

Within the current regulatory frameworks on waste management in both Spain and Norway, there have not been identified any significant challenges with the valorisation of uncontaminated washing residues and their use as secondary raw materials. Despite their status as a waste, the utilisation of these materials is not found restricted by their classification but rather by the availability of cost-effective treatment and utilisation options. However, the use of secondary materials requires compliance with technical standards and specifications applicable to the raw materials used for the same purpose. By aligning the treatment processes for waste such as sludge with relevant product legislation and standards, these materials have the potential to be utilised for higher-value applications. For instance, fillers and SCM must comply with standards for water demand and reactivity levels for their use in concrete, LWAs must meet specific criteria in terms of density and thermal properties for lightweight construction materials, and 3D printing mixes must comply with requisites for rheological behaviour and controlled shrinkage. Compliance with these standards is crucial for gaining market acceptance and ensuring that the secondary materials can be used in manufacturing processes of construction products as an equivalent alternative to virgin materials.

With the presence of contamination, as is the case for the washing residue from the treatment of polluted excavated material at Velde in Norway, further consideration needs to be taken to ensure that the impact on human health and the environment under product legislation does not exceed the impact under waste legislation. According to Norwegian legislation, the contaminated washing residue must be treated by an approved waste management company, complying with requirements in the landfill directive. Appropriate treatment processes must be developed and implemented to reduce or eliminate contaminants and/or their release if these materials are to be valorised, ensuring that the final product meets safety and environmental standards applicable to its intended use. Establishing clear EoW criteria for waste materials not only determines a process for compliance with product legislation and standards but also provides the necessary documentation to ensure confidence in using these secondary materials. This documentation is crucial for assessing and mitigating the risks to human health and the environment when the material is used as a product.

### 2.4. Adaptation of the EoW Framework for Specific Valorisation Pathways

While the EoW concept provides a robust framework, it may not always be feasible to apply it uniformly across all waste streams or contexts due to its complexity and the time required for implementation. In the context of valorising certain types of mineral residues such as sludges and tailings, developing EU-wide criteria may not be necessary. The study cases illustrate this point. Provided that the waste material from Velde and Hormisoria can be technically validated for use as fillers, SCM, LWA, or in 3D printing, the secondary materials are typically intended for sale at a local/national market. This localised application diminishes the need for harmonised EU-wide criteria. Instead, using the EoW framework as a guideline can help outline the steps needed to meet the requirements of specific valorisation pathways on a national level. This approach would ensure compliance with local regulations, economic viability, and alignment with market demands, as well as the minimisation of negative environmental effects. Even though criteria based on the framework would be developed for case-specific valorisation pathways applicable to the respective producers, it could serve as a foundation for developing national or EU-wide EoW criteria for selected waste streams. Based on the insights and learnings from these local cases, guidelines can potentially be created to address local regulatory and market conditions. This would provide a scalable model that can be adapted and expanded to meet broader regulatory requirements across different jurisdictions.

An important aspect of valorising secondary materials is the market situation and the feasibility of selling these materials. The framework of the EoW criteria includes determining whether a market or demand exists for a specific substance or object. However, in practice, a market may not always exist before developing new valorisation pathways for materials not currently utilised. New valorisation pathways can, however, create new markets for specific secondary raw materials. By demonstrating that the valorised washing residues can be put to useful purposes such as fillers, SCM, LWA, and 3D printed construction materials, and thus are less likely to be discarded, the principles of resource efficiency and circular economy are supported. To determine the potential for these applications, it is essential to evaluate the demand for these secondary raw materials in the construction industry. This evaluation should explore whether there are construction companies or other entities interested in buying and using these materials.

Ultimately, the successful valorisation of washing residues hinges on both the technical feasibility of producing high-quality secondary products and the economic viability of finding or creating a market for these products. This economic feasibility can depend on local conditions such as the availability of virgin raw materials and regional industry demand. For example, if local industries demand specific secondary raw materials and there is a limited supply of virgin materials, the economic feasibility of valorising waste materials increases. The development of EoW criteria plays a crucial role in this context by providing a clear regulatory framework that helps to standardise processes and ensure compliance with relevant legislation.

## 3. Materials and Methods

### 3.1. Washing Sludge at Site in Norway

**Figure 1:**
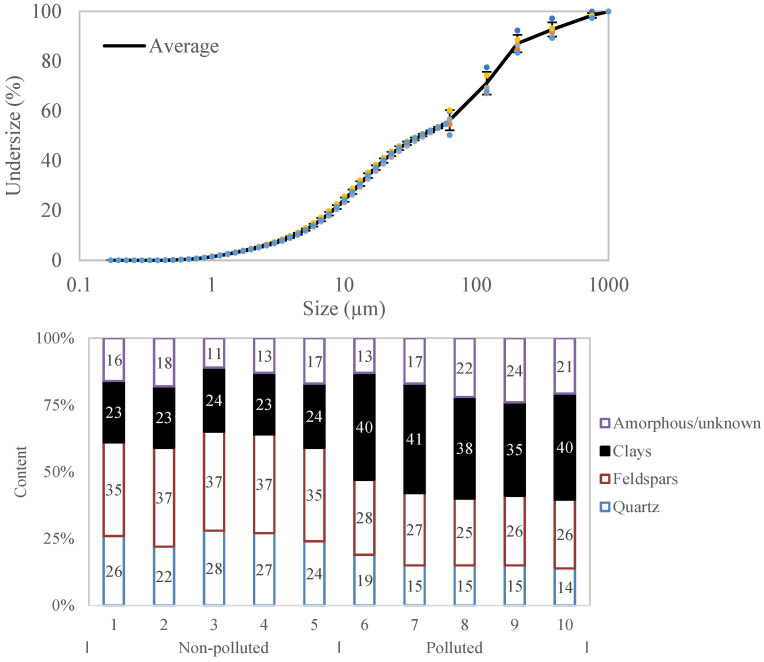
Properties of Norwegian sludges: **top**: particle size distribution (the coloured dots show five replicate measurements); **bottom**: mineralogical composition.

Aggregate-washing sludge from the production of recycled aggregates from demolition and excavated material was sampled at the production site (Velde, Norway). A total of 10 samples were collected during different times of the year. These washing sludges are sometimes contaminated with inorganic oil and additional clayey material. Two waste streams, polluted and non-polluted, were identified during this campaign. The contamination increases the content of organic phases and clay in the samples, but no significant health concerns were identified for any of the samples. Organic phases may potentially affect the hydration development of cement phases. Therefore, the non-polluted samples were used for the development of the valorisation paths proposed. The variability of the properties of the materials (non-polluted material: samples 1 to 5, polluted material: samples 6 to 10, Figure 1, bottom) within each waste stream was within acceptable limits and they can be regarded as two types of waste material to be processed.

The samples were dried, deagglomerated in a mill, and characterised, including XRD with Rietveld analysis, and PSD by laser diffractometry and sieve analysis. The disc mill only grinded the material at low energy to disaggregate the agglomerated material and not to engineer the particle size distribution. Figure 1 (top) depicts the particle size distribution (determined by sieving coupled with laser diffractometry for particles < 90 μm) and mineralogical composition (determined by a Rietveld analysis of XRD patterns) of the fillers. Due to the limited grinding, the particle size distribution is unchanged from that of the on-site sludge. Most of the material corresponded to particles < 0.063 μm, but some contamination with coarser particles was also noted. The size range impedes complete classification as a filler or as a sand. For the purpose of this research, the material was treated as a filler because most of its mass is in that particle size range. The main crystalline phase groups are quartz, feldspars (albite and microcline), and clays (mostly illite, but also kaolinite and clinochlore). The average density and loss on ignition at 1050 °C of the material were (2.64 ± 0.02) g/cm^3^ and 1.86 wt%, respectively.

The clay content consists of a blend dominated by illite and also includes kaolinite and clinochlore at lower proportions. The two most important features of these clay phases are their water demand when included in fresh cementitious mixes, and their capability to be thermally activated to increase their reactivity. The total content of clay is, however, in the range of 23–24% for the non-contaminated material, and of 38–41% for the polluted material. This range of contents is quite challenging, as it is relatively high for the use as filler, and relatively low for the use as supplementary cementitious material. The clay content of the non-polluted material and polluted material make them more suitable for applications as filler and as supplementary cementitious materials, respectively.

#### 3.1.1. Application as Filler

For testing the performance as filler in the production of self-compacting mixes, optimisation at paste and mortar level was first conducted, and then self-compacting concrete mixes were produced and optimised. The used materials were modified polycarboxylic ether-based superplasticiser (MasterGlenium 51^®^, Master Builders Solutions, Mannheim, Germany), cement CEM I 52.5 N, standard CEN sand, and fillers produced with the secondary sludge.

First, the optimal water-to-powder ratio (W/P) in pastes for each of the five non-polluted samples was determined as recommended in [1]. Pastes with sludge filler (30 to 60 wt% of the powder) were tested for water-to-powder volume ratios (W/P) of 1.2, 1.3, and 1.4. Following that, the superplasticiser content was adjusted in mortar mixes to achieve slump-flow values in the range of 240–260 mm (=relative slump-flow, Γ_p/m_, between 4.76 and 5.76), reducing the water content to between 85 and 95% of βp so that the targeted V-funnel time (EN 12350-9, adapted for mortar and using a small V-funnel) was in the range of 7–11 s. These mortar mixes were designed with the content of sand representing 50% of the mortar volume and optimised filler content, superplasticiser dose, and W/P.

**Figure 2:**
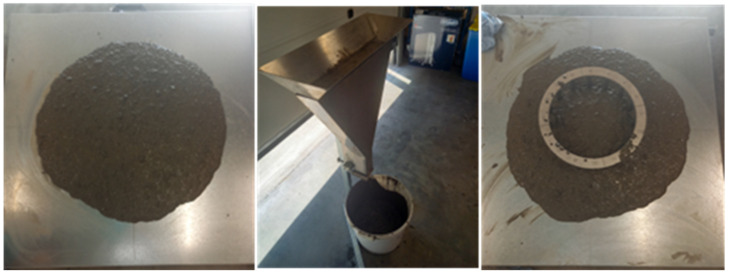
Fresh tests of optimised self-compacting mixes containing sludge-based filler.

Once the matrix system was optimised, six batches of self-compacting concrete were prepared with filler and cement contents of 170–180 and 356 kg/m^3^ of concrete, respectively, and a maximum aggregate size of 20 mm. Fresh and hardened properties were tested (Figure 2), including V-funnel, T_500_, and slump flow. In the hardened state, compressive strength, saturated resistivity, chloride migration, and carbonation rate were measured.

#### 3.1.2. Application in the Production of SCM

The substantial content of clay minerals in the aggregate-washing sludges impeded their excellence as filler but might be beneficial for the use as SCM. The activation of clay minerals via calcination is an established route for SCM production. A screening of the process conditions was carried out by first testing a wide range of temperatures: 600–1000 °C. Calcination was carried out in a box furnace (3 °C/min heating and cooling, 1 h dwell time). The reactivity of aggregate-washing sludges was measured using isothermal calorimetry in a TAM Air on an R^3^ paste (ASTM 1897-20 [32]). This test measures the heat release during 7 days at 40 °C of a mixture of the SCM with Ca(OH)_2_ and minor contents of K_2_SO_4_, KOH, and CaCO_3_ to simulate the reaction in a cementitious material. The heat release during the reaction of this paste is the measure for reactivity.

Further on, as initial optimisation pathways for increasing the reactivity of the samples, milling and co-calcination with Ca(OH)_2_ were considered. The particle size distribution of the sludges, presented in Figure 1, is larger than most contemporary SCMs. Therefore, the R^3^ heat release was repeated on samples after ball milling to a d_50_ of 8–10 µm.

The presence of Ca was theorised to cause the substantial reactivity in calcined dredging sediments [16]. The co-calcination of the samples with 5 wt% of Ca(OH)_2_ was therefore carried out, combined with milling to d_50_ 8–10 µm.

#### 3.1.3. Application in the Production of LWA

The combination of quartz and feldspars in sludges with a granitic origin promises a good match with the compositional requirements of the bloating process, needing only minor correctional additives [33,34]. Granitic sludges have therefore been studied successfully at lab scale, but more in-depth knowledge needs to be obtained on their melting behaviour to fully assess their potential use in an industrial bloating process. The thermodynamic equilibrium phase composition is modelled for the available compositions of aggregate-washing sludge using the Equilib module of FactSage 8.0 and the FactPS and FT Oxid databases. The modelling was carried out at temperatures of 900–1400 °C, enabling the study of the evolution of the wt% melt with temperature. Previous work [35] showed that this approach provides an accurate estimation of the actual melting behaviour and a more accurate assessment of the suitability of a material than the conventional compositional checks according to Riley [18] or Cougny [36].

A further validation of these simulations was performed via lab-scale experiments. The experiments were carried out by granulating aggregate-washing sludges in an Eirich mixer and bloating in a box furnace (3 °C/min heating and cooling, 1 h dwell time). In increments of 25 °C, the temperature was increased while investigating the microstructure of the granules using SEM and evaluating the extent of sticking to the crucible. The absence of stickiness to the crucible and presence of bubble-shaped pores indicates suitability for bloating [35].

### 3.2. Washing Sludge at Site in Spain. Application in 3D Printing in Construction

**Figure 3:**
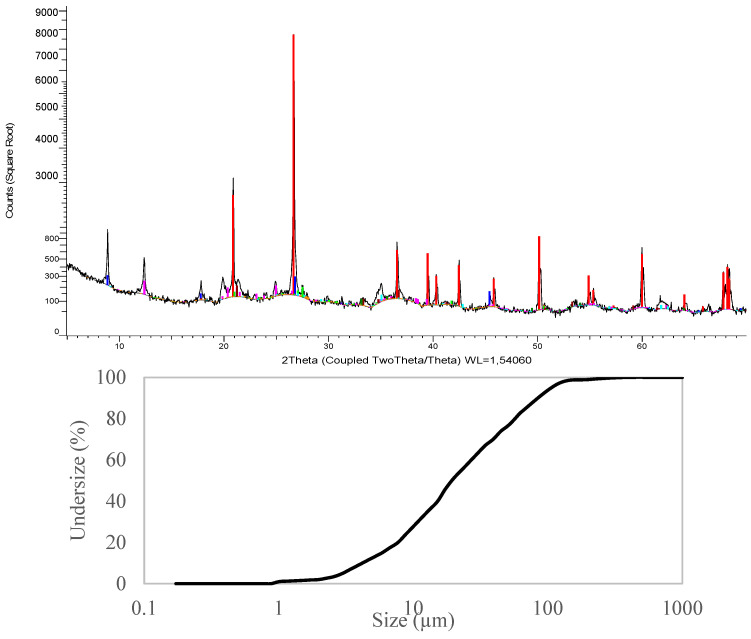
Properties of Spanish sludges. Top: particle size distribution; bottom: mineralogical composition (DRX pattern with identified phases: quartz (red), muscovite (blue), microcline (light green), kaolinite (pink), goethite (brown), hematite (green), clintonite (light blue), anorthite (orange)).

In terms of clay characterisation, the physical and chemical properties of the clay sludge (Hormisoria, Soria, Spain) were determined. This detailed knowledge forms the basis for a precise assessment of the clays in 3D printing applications, revealing any required adaptation in the design of the mix due to specific geographic and environmental conditions. The particle size distribution and XRD analyses are presented in Figure 3. The composition was predominantly quartz (44%), clays (41%, confirmed by sedimentology analysis, including illite and kaolinite), feldspars (3%, including microcline), and other (12%, including clintonite, goethite, hematite, and dolomite, and poorly crystalline). The density of the material was 2.62 g/cm^3^. Due to the high clay content, the plasticity index was 21.4, which makes the material very suitable for the rheological properties required for 3D printing.

A critical aspect of the formulation of a mortar for 3D printing is ensuring the necessary cohesion. The content of clays contributed to this cohesion, but the sludge still required a commercially available aggregate subproduct. This aggregate was selected to compensate the sludge to achieve the desired particle size distribution curve that aligns with the standards defined for sands with 0/2 designation. While the resulting mortar may not reach strengths comparable to other more conventional types of mortars, the final outcomes were considered more suitable for the specific 3D application in terms of workability.

The combination of the clay characterisation of the sludges and mortar development opened opportunities for innovation in construction by 3D printing. The dosage was adjusted to achieve the most porous structure possible, facilitating the drying speed of the mix and, therefore, reducing the time for its implementation. After the conducted tests, it was observed that limestone fine aggregate may be needed to provide a sufficient framework to meet this requirement. Moreover, in view of significant retraction development during drying, vegetable fibres were considered to increase the cracking resistance capacity of the compound without compromising its strength, as the fibres had a relatively small diameter. Lastly, enzymes were considered as additives to contribute to the cohesion of the overall mixture, enhancing the compressive strength of the hardened mortar. Water was added on demand to achieve the necessary consistency (varying in the range of 30–40 wt%).

The fresh mortars were tested for three crucial properties: consistency (ability to maintain their shape and resist deformation under load), extrudability (ability to be extruded uniformly and controllably through a nozzle), and buildability and drying rate (capacity to maintain their form and structure during the construction process, as well as the speed at which the mortars achieve the desired mechanical strength). These fresh-state tests were essential for understanding the mortar’s behaviour during the initial application phase and for adjusting the formulation as needed to achieve optimal properties in 3D printing.

In the hardened state, mechanical characterisation included compression and tensile tests. Drying shrinkage was determined in prisms of 40 × 40 × 160 cm^3^.

For the proper development and characterisation of a material intended for the manufacturing of parts through additive manufacturing, various extrusion tests were conducted throughout the material development process. These include manual extrusion tests that were performed in the laboratory with a manually operated mortar gun, and implementation in a small-format 3D printer. Once the optimal formulation was developed, applicability tests were performed as a proof of concept and practical validation. These tests involved the actual extrusion of the material under conditions simulating the additive manufacturing process. Aspects such as consistency, layer adherence, structural stability, and other crucial properties for the final quality of printed parts were evaluated.

## 4. Results

### 4.1. Application as Filler

Concerning the effects on the consistency, the relationships between the relative slump-flow values and the W/P for various filler contents with respect to total powder (P30 to P60 for 30 to 60 wt% of filler) were described in previous work [37]. Water demand remained unaffected for low filler contents. This result can be attributed to the fact that, at low filler ratios, the dilution effect of cement by the sludge-based filler was compensated by the relatively high content of clays in the sludges. The demand for water may eventually limit the amount of filler that can be incorporated in the mixes to achieve sufficient self-compactability at a competitive cost and with adequate performance. The slump-flow values as a function of the W/P demonstrated that the filler content can reach values as high as 40 wt% of the total powder content. Higher filler contents would require the additional treatment of the sludges to reduce the content of clay (which may be valorised with a different approach, e.g., as calcined clays in the application as SCM).

**Figure 4:**
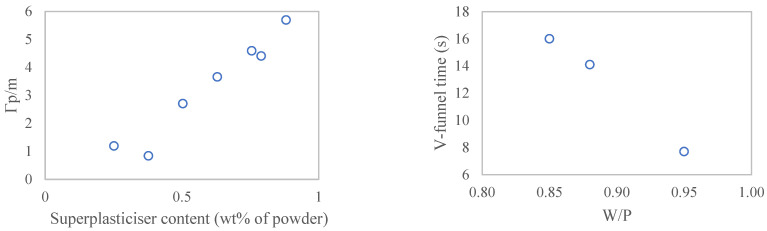
Optimisation of superplasticiser and water content for target slump-flow (**left**) and V-funnel time (**right**), respectively.

Figure 4 shows the optimisation of the superplasticiser content for mortar mixes with the sludge-based filler (W/P = 0.85 and 40 wt% filler content). The relative slump-flow is a linear function of superplasticiser content (indicating a range below the saturation point). The targeted slump-flow was achieved for 0.88 wt% of (the solid content of) superplasticiser relative to powder (Figure 4, left). However, the viscosity of the mix was still excessively high, as demonstrated by the V-funnel time. The W/P had to be further increased from 0.85 to 0.95 to achieve the required V-funnel time (Figure 4, right), and to maintain the slump flow value, this increase in water content was compensated with a reduction in the superplasticiser from 0.88 to 0.66 wt%.

The results from fresh mortar mixes show that, even though the filler has a water demand level which is higher than desired, it also contributes to the viscosity of the mix. This means that these self-compacting mixes have greater cohesion than mixes with other materials used for the same purpose (e.g., limestone and fly ash). In practice, this would mean less segregation and the ability to design self-compacting concrete with a higher coarse aggregate content.

For the mortars that met the target slump-flow and V-funnel time, hardened properties were determined: compressive strength and porosity. The properties of the mortars for the five non-polluted sludge-based fillers (filler content = 40 wt%, superplasticiser = 0.66 wt%, and W/P = 0.95), demonstrated an average compressive strength of the mortars of 61.5 ± 2.6 MPa and an average porosity of 13.3 ± 0.3%, indicating a very promising performance considering that the effective water-to-cement ratio of the mixes was 0.5.

The fresh properties of the self-compacting concrete demonstrated a V-funnel time of 8–9 s, T_500_ of 3–4 s, and a slump flow of 600–750 mm. These results in the fresh state were compliant with the requirements.

In the hardened state, the use of sludge-based filler instead of limestone filler showed no noticeable effects on the performance of the self-compacting concrete. Compressive strength was maintained at 51.6–52.4 MPa, saturated resistivity at 13.5–14.3 kΩ·cm, chloride migration coefficients at 8.3–8.9 10^−12^ m^2^/s, and carbonation rate at 0.60–1.15 mm/days^0.5^. The carbonation rate was the property that showed the most sensitivity to the use of the sludge-based filler. One possible reason for this is the indirect effect of increased cohesion and reduced bleeding, but the reasons for this are not clear and require further research.

### 4.2. Application in the Production of SCM

**Figure 5:**
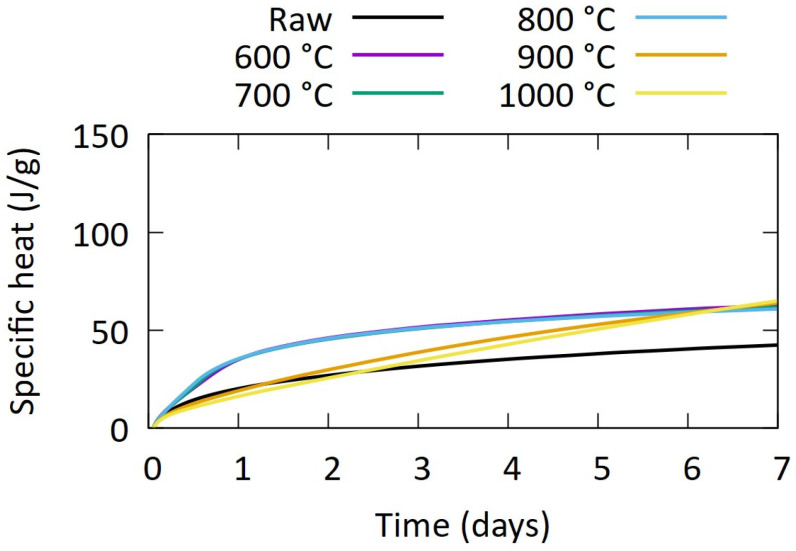
Heat release during R^3^ test on (calcined) aggregate-washing sludges.

The reactivity of the calcined washing sludges is shown in Figure 5 for the different calcination temperatures. Overall, the calcination treatment has slightly increased the reactivity in comparison with the raw sample, which reached 42 J/g after 7 days. However, the observed heat after 7 days of 61–65 J/g SCM of the calcined samples is low, especially considering that the dredged sediments from Snellings et al., reached 250–270 J/g after 7 days with a lower clay content [16]. The heat after 7 days is not dependent on the calcination temperature, but the shape of the curve, and thus, the heat after 1–3 days changes significantly between ≤800 °C and ≥900 °C. Whether this has to be attributed to sintering or a change in phase composition has not been clarified at this stage.

**Figure 6:**
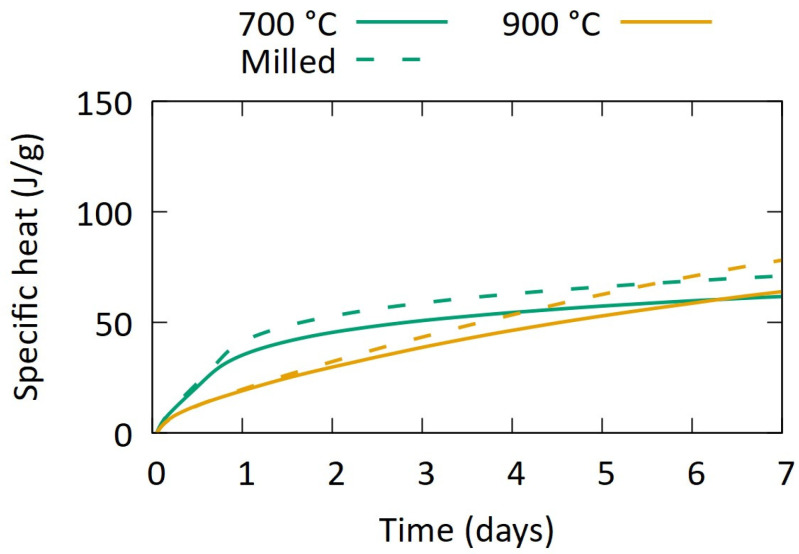
Heat release during R^3^ test on (calcined) aggregate-washing sludges after milling to d_50_ 8–10 µm.

Figure 6 shows that milling successfully increased the reactivity of the samples, independent from the calcination temperature. However, a substantial gap between the absolute values and the desired reactivity of an SCM remains.

**Figure 7:**
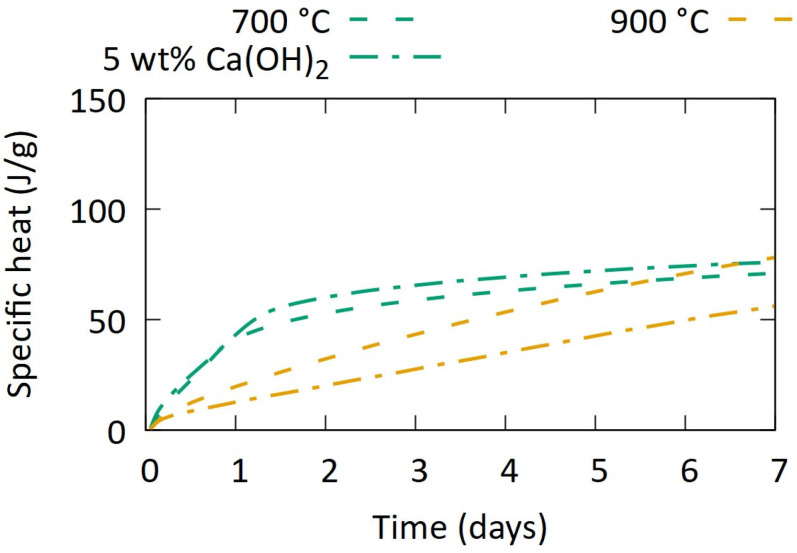
Heat release during R^3^ test on aggregate-washing sludges co-calcined with Ca(OH)_2_ after milling to d_50_ 8–10 µm.

The R^3^ heat release in Figure 7 shows that the reactivity with an added source of calcium again slightly increased for the samples at 700 °C, while at 900 °C a significant negative effect is observed. The difference with the results from Snellings et al. [16] is probably the flash-calcination there vs. soak calcination here and future work should study the effect of heating/cooling rates and residence time to properly document and understand the phenomena at play.

### 4.3. Application in the Production of LWA

**Figure 8:**
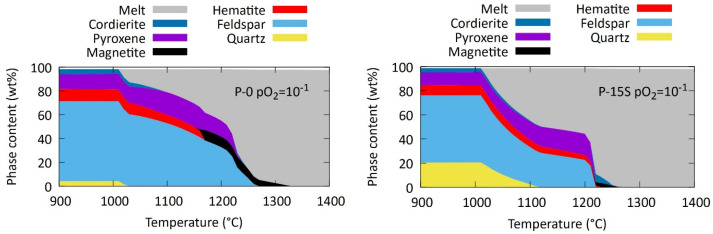
Simulated equilibrium phase composition of aggregate-washing sludge (P-0, **left**) and aggregate-washing sludge with the addition of 15 wt% SiO_2_ (P-15S, **right**).

Figure 8 shows the equilibrium phase composition at 900–1400 °C for one of the studied aggregate-washing sludges and the sludge with a 15 wt% addition of SiO_2_. Melt formation already starts at 1010 °C due to the eutectic melting of quartz and feldspars. The melting of the quartz and feldspars gradually proceeds, until a drastic increase in melt is observed due to the melting of pyroxenes. Previous work showed that bloating is ideal with about 50 wt% of melt. For process control in industry, the wt% of melt thus needs to be stable as a function of temperature around 50 wt% of melt. This stability is substantially improved with the addition of 15 wt% of SiO_2_, while the increase in melt is rather steep for the composition of the sludge as received.

**Figure 9:**
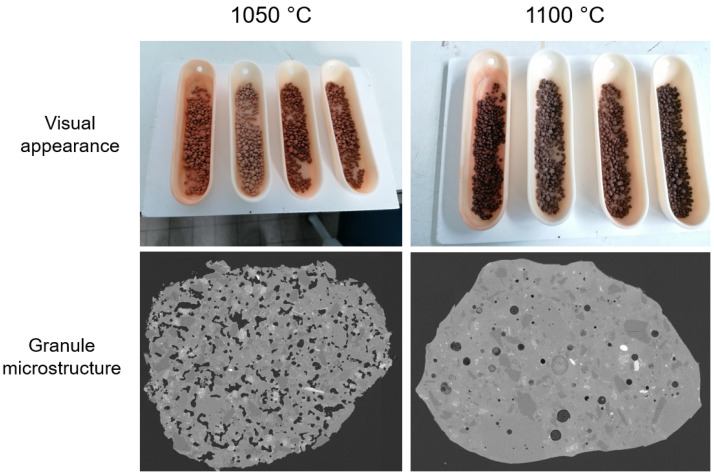
Visual appearance and SEM microstructure image of bloated aggregate-washing sludges.

Figure 9 illustrates that the desired behaviour with an absence of stickiness to the crucible and presence of bubble-shaped pores is achieved at 1100 °C. Unexpectedly, also without the addition of quartz (P-0 in Figure 8, left), the desired microstructure could be achieved without excessive sticking to the crucible. Future work should reveal whether these conclusions hold when upscaling the process in a rotary furnace.

### 4.4. Application in 3D Printing in Construction

Table 1 summarises the main dry components of the final dosage used for 3D printing applications.

**Figure 10:**
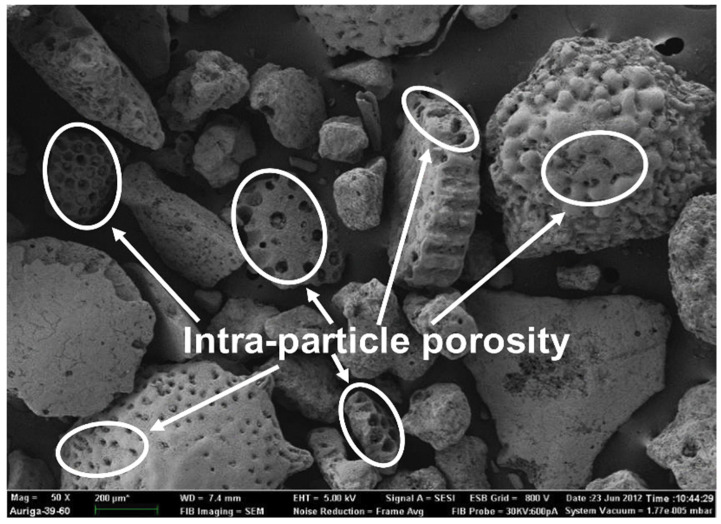
Electron microscopy of the aggregates added to 3D printing mixes, showing their porosity.

The effects of the use of limestone in the mortar formulation were mainly the contribution to a faster drying and the stabilisation of the mortar. After mixing with the clayey soil, the porous aggregate absorbs water (Figure 10), drastically reducing the free moisture in the soil. Moreover, the distribution of charges on the surface of the clay soil particles is modified, resulting in an ionic exchange between the sodium (Na) in the soil and the free calcium (Ca) in the limestone. The effect is that the clay partially loses its ability to retain water. The aggregates and the addition of chamotte are intended to accelerate the drying or availability of water for mixing the clay, increasing the speed of hardening and therefore the speed at which it can be placed on site and loaded. In the mid-term, the provision of a calcium source provides a potential pozzolanic reaction with the clays, thereby increasing the strength and frost resistance of the mortar.

The use of vegetable oil can also improve the malleability and workability of the mortar, as well as weather resistance. Oil admixtures can also reduce shrinkage. The plant fibres, such as esparto grass or jute, provide an ideal cracking resistance. Given their rough surface, the fibres demonstrated efficient adherence to the matrix. Once the material has dried, its performance is improved by the vegetable fibres, while the clay matrix provides protection against fluctuations in relative humidity that can trigger the degradation of the vegetable material.

In the initial experimental phase, linear shrinkage in the longitudinal direction was around 3.5%, while linear shrinkage in the transverse direction was 7.5%. In the final dosage, these values were reduced to 2% and 3.75%, respectively. The mechanical tests resulted in 1.5 MPa and 0.6 MPa for the compressive and tensile strength, respectively. Additional adjustment may be necessary to adjust the strength to the desired application. Overall, these values are considered suitable for the application in additive manufacturing.

**Figure 11:**
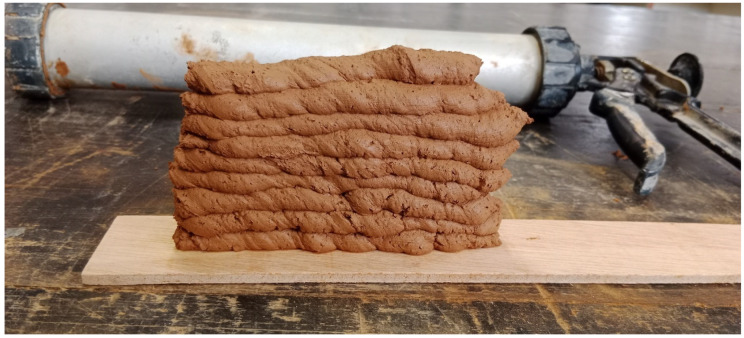
Example of mortar gun extrusion test.

**Figure 12:**
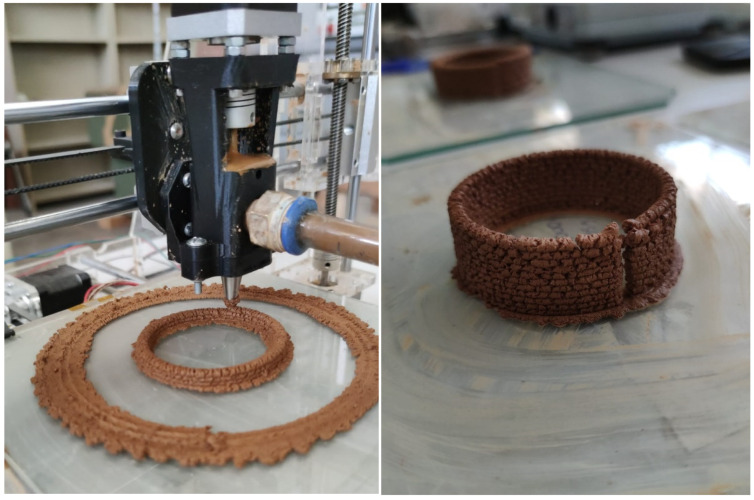
3D printing tests for the optimisation of the formula.

Figure 11 shows the result of the manual extrusion tests. These tests offered a good practical approximation of the material’s behaviour during the additive manufacturing process. Figure 12 shows the result of the implementation with the small-format 3D printer. These tests provide a preliminary understanding of the potential of the developed material for use in large-format additive manufacturing.

**Figure 13:**
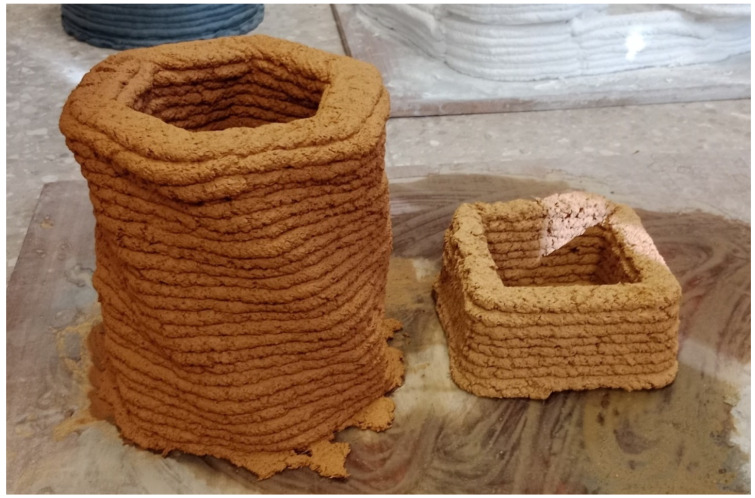
Additive manufacturing pieces printed with the new mortar tailored from clayey sludges.

The results obtained from the additive manufacturing simulation are highly favourable, demonstrating that the developed material is optimal for use in additive manufacturing processes. The ability to print organic and complex shapes highlights the versatility of the developed clay mortar in 3D printing applications (Figure 13).

## 5. Discussion

Valorisation options for transforming aggregate-washing sludges and tailings into construction materials were presented as a step toward promoting circular economy principles in mineral-extracting industries and enhancing the sustainability of the construction industry. Such high-value applications include the use as inert fillers for concrete, supplementary cementitious materials (SCMs), lightweight aggregates (LWAs), and 3D printing applications. The comprehensive characterisation of these materials and results from preliminary tests provide a solid proof-of-concept for supporting their implementation as constituents of construction materials. However, continued experimentation is necessary to provide sufficient data for statistical analyses and define the ranges of variability for the performance across specific applications. Considering the targeted applications, such statistical analyses seem more valuable once the technologies are upscaled, so that the influence of industrial production is captured. Specific considerations are suitable for each specific application, as follows.

### 5.1. Inert Fillers for Concrete

The use of aggregate-washing sludges as inert fillers in self-compacting concrete (SCC) demonstrated promising results. An initial concern was that the high clay content in the sludge can increase the water demand and the need for superplasticisers. However, the clay content in the sludges produced a much milder effect than those reported in [7,8]. For the specific sludges under study, it seems that this is not a major limitation when clay contents are not higher than 40% in the sludge, and the sludge is not reaching more than 40% of the total powder content in the matrix. The clay content also contributed to the viscosity of the mix, enhancing its cohesion and reducing segregation and bleeding risks, particularly concerning aspects for fluid cement-based mixes. Self-compacting mixes could be optimised to meet the performance criteria in terms of compressive strength and durability properties. In this sense, the effects of aggregate-washing sludges (containing clay impurities) are quite different from the effects of industrial sludges in terms of the rheological properties of the resulting concrete mix [38,39,40,41]. Mineral wastes have advantages over more reactive powders (e.g., silica fume, metakaolin, and even Portland cement) and limestone powder in terms of limited segregation and bleeding in self-compacting concrete [4,5]. One possibility for explaining this is that the use of milled wastes increases viscosity and reduces the dependence on viscosity modifiers to produce stable self-compacting mixes. For the case of aggregate-washing sludges, the effects are not only connected to the particular particle size distribution, but also to the chemical composition of the involved clays [8]. Further optimisation and cost analysis are necessary for each specific site to ensure economic feasibility in view of competing fillers and local demand for SCC.

### 5.2. Supplementary Cementitious Materials (SCMs)

The potential of aggregate-washing sludges to be used as SCMs was assessed through calcination processes aimed at enhancing their reactivity. Various clay minerals (e.g., illite, kaolinite, and clinochlore) show differences in their reactivity after thermal activation [16,42,43,44,45]. The reactivity after the thermal treatment of clay-containing sludges will be connected to the clay types and contents in the material. The reactivity levels achieved in this study were below the desired thresholds, indicating the need for further refinement. Techniques such as increasing the heating/cooling rates and exploring alternative thermal activation methods like flash calcination were identified as potential avenues for increasing reactivity. Additionally, clay concentration techniques or co-calcination with other pozzolanic materials or additives could further enhance its performance as an SCM. Such additional processing would increase processing costs, which may be well justified if high reactivity levels are demonstrated.

### 5.3. Lightweight Aggregates (LWAs)

The production of LWAs from aggregate-washing sludges involves the sintering of the sludge to form porous, lightweight particles. The chemical composition of most washing sludges, rich in quartz and feldspars, is suitable for the bloating process, requiring minor additions of SiO_2_ to improve the stability of melt formation. The experimental validation showed promising results with the formation of rounded pores, indicative of sufficient melt formation at higher temperatures without granule collapse. This application demonstrates a high potential to decrease the environmental impact of LWAs (increased circularity and reduced energy demand with production at lower temperatures). However, further research is needed to optimise pore volume and confirm the temperature windows for the sintering process in industrial settings. The potential added value with respect to applications of washing sludges as inert filler or SCM still requires a cost analysis, but it seems that from a technical point, their application in LWA is highly convenient. The clay content does not play a limiting role in the design of the processing, and the potential incidence of the presence of minor contaminants (mineral oil and heavy metals) may well be reduced with the encapsulating ability of the sintering process.

### 5.4. 3D Printing Applications

The application of aggregate-washing sludges in 3D printing mortar development represents a novel and innovative use of these materials. A high clay content in the sludges contributes to the necessary rheological properties for 3D printing (cohesion and extrudability). Common corrections in the formulation of 3D printable mortars are still necessary by adjusting the water content, superplasticisers, and other additives to achieve the required consistency and extrudability and promote drying and hardening, as well as increasing compressive strength.

The upscaling of this technology requires future efforts to assess the buildability of the optimised mix. The buildability of the mix plays a crucial role as it determines the maximum printing rate and the minimal time between the deposits of two successive layers. Adaptations to increase the strength gain rate may be needed to reach printing rates that are more adequate with the scale of construction on site. Curth et al. [46] suggest that full-scale printing with earthen mixes (comparable to our sludge-based mix) may require angled printing with a low feed rate and minimal water content. This could potentially reduce the competitive advantage of 3D printing as a building technology. Perrot et al. [47] indicated that alginate as a biomaterial that can be used instead of carbon-intensive cement to increase the buildability of the mix. The compatibility of the sludges under study with alginate is worth investigating in future research.

## 6. Conclusions

Technical feasibility for the valorisation of washing sludges as inert filler, for self-compacting concrete production, for the production of lightweight aggregate, and as a precursor for 3D printing mortar was supported by experimental results. Additional piloting studies to determine the economic feasibility (tied to the cost of processing these materials and the market demand for the end products), and life cycle assessments, would contribute to the subsequent cost–benefit analyses that would fully depict the economic and environmental implications of these valorisation strategies for specific exploitation sites.

Among the remaining technical challenges, secondary materials such as washing sludges and tailings require that potential variability in composition is specifically addressed with statistical analyses (indicating the potential need for additional control and homogenisation processing techniques to ensure uniform performance in construction applications). For the sites under study, the variability in composition and properties of the sludges was very limited, allowing the use of these studied materials without the need for additional homogenisation.

For the applications as inert filler and thermally activated SCMs, the clay content poses a challenge. In the first case, it is too high and increases water and superplasticiser demands. This can, however, be compensated by a higher cohesion, which is beneficial in the production of more robust self-compacting concrete. In the second case, it is too low and results in insufficient reactivity to justify the thermal processing. The applications in the production of LWAs or 3D printing mixes appear as the two most straightforward alternatives. Advanced separation techniques may be interesting to the degree that they may optimise the clay content in the sludges to the target values that are more convenient for the applications as filler and SCMs, allowing fine-tuned valorisation channels.

Transforming washing sludges to valuable secondary raw materials depends on effectively navigating regulatory, technical, and market considerations. Developing robust End-of-Waste (EoW) criteria tailored to the specific valorisation pathways and regulatory landscapes (in this case Spain and Norway) can facilitate the integration of washing sludges into the circular economy. This involves ensuring compliance with technical standards, evaluating the material’s properties, ensuring the removal of harmful contaminants, and verifying that the end use does not pose risks to human health or the environment. By aligning these criteria with local market demands and regulatory contexts, progress can be made toward realizing the potential for innovative and sustainable applications of washing sludges in construction.

## Figures and Tables

**Table 1:** Main dry components of the final dosage used for 3D printing applications.

Component	Nature	Percentage in Weight
Limestone aggregate	AF-0/2-C according to UNE146091	60%
Clay soil	Sludge	25%
Additive	Enzymes	0.3%
Additive	Vegetable fibres (length of 1 cm)	1%
Additive	Chamotte 0/2	13.7%

## Data Availability

The data presented in this study are available on request.
